# Barriers and facilitating strategies for healthcare access and reception for transgender children and adolescents

**DOI:** 10.1590/0034-7167-2024-0266

**Published:** 2025-03-17

**Authors:** Juliana Cristina Pereira Silveira, Danton Matheus de Souza, Clarissa de Souza Cardoso, Márcia Aparecida Ferreira de Oliveira

**Affiliations:** I Universidade de São Paulo São Paulo São Paulo Brazil Universidade de São Paulo. São Paulo, São Paulo, Brazil; II Universidade Federal de Pelotas Pelotas Rio Grande do Sul Brazil Universidade Federal de Pelotas. Pelotas, Rio Grande do Sul, Brazil

**Keywords:** Transgender Persons, Sexual and Gender Minorities, Child, Adolescent, Barriers to Access of Health Services, Personas Transgénero, Minorías Sexuales y de Género, Niño, Adolescente, Barreras de Acceso a los Servicios de Salud

## Abstract

**Objectives:**

to identify barriers and strategies that facilitate access and welcoming transgender children and adolescents in the Healthcare Network.

**Methods:**

an integrative literature review, with articles, available in full, with children and adolescents between 6 and 19 years of age, published between January 2013 and April 2023, in Portuguese, English or Spanish. The level of evidence was assessed using the JBI classification.

**Results:**

the main barriers were low professional knowledge, hostile professional interactions, and sexual presumption. Facilitating strategies included continuing education, questioning about gender and pronouns, and encouraging a respectful and welcoming environment.

**Conclusions:**

this study indicates numerous barriers to access and support for transgender children and adolescents and strategies that can transform this context, aiming to move towards comprehensive care that recognizes, validates and supports gender identity with appropriate, safe and inclusive care.

## RESUMEN

**Objetivos:**

identificar las barreras y estrategias facilitadoras para el acceso y acogida de niños y adolescentes transgénero en la Red de Atención a la Salud.

**Métodos:**

revisión integradora de la literatura, con artículos, disponibles íntegramente, con niños y adolescentes entre 6 y 19 años, publicados entre enero de 2013 y abril de 2023, en portugués, inglés o español. El nivel de evidencia se evaluó mediante la clasificación del JBI.

**Resultados:**

las principales barreras fueron el bajo conocimiento profesional, las interacciones profesionales hostiles y la presunción sexual. Las estrategias facilitadoras fueron educación continua, preguntas sobre género y pronombres y fomento de un ambiente respetuoso y acogedor.

**Conclusiones:**

este estudio señala numerosas barreras para el acceso y la atención de niños y adolescentes transgénero y estrategias que pueden transformar este contexto, con el objetivo de avanzar hacia una atención integral que reconozca, valide y acoja la identidad de género con una atención adecuada, segura e inclusiva.

## INTRODUCTION

Transsexuality refers to a person who does not identify their gender with the sex they were assigned at birth. The term “trans” is widely used to encompass this population, which demands social recognition^(1)^. Thus, trans women are those who were born male but identify with the female gender; trans men are those who were born female but identify with the male gender; and gender non-binary people (GNB) are those who feel that their identity is outside or between the female and male identities, identifying with both or neither^(1,2)^.

This identification process emerges gradually during childhood, beginning in early childhood. By the end of childhood, a child is already aware of the definition of their gender. However, gender identification may not be mentioned due to the social representation of cisgender and the stigmatization of transsexuality, and may be mentioned in adolescence^(3)^. It is worth noting that recognizing the existence of transgender children and adolescents means defending their needs and desires as human beings and subjects of rights, based on a claim for gender expressiveness that is different from cisgenderism^(3)^. In recent years, there has been an increase in studies related to the transgender population, but with a focus on adults. Few studies have focused on children and adolescents, and those that do exist have focused on the repercussions of transphobia on their mental health^(4,5)^.

It is estimated that the proportion of individuals who identify as transgender worldwide is between 0.1% and 2% of the general population^(6)^. In Brazil, a study that assessed the proportion of gender diversity showed that, of 5,930 people over 18 years of age, 1.9% have gender diversity, of which 0.7% are transgender and 1.2% are GNB. Furthermore, it is estimated that around 1,090,200 adults may identify as transgender and 1,880,200 as GNB, thus totaling almost 3,000,000 people with gender diversity^(1)^. In the child and youth stages, the literature still diverges, with an average between 0.1% and 1.3%^(6,7)^. However, these studies may not be accurate in terms of the proportion of transsexual individuals, especially with children and adolescents, since identification is masked in a context of stigma and invisibility of child and adolescent transsexuality^(1,3,8)^.

Recently, in order to advance this issue, the World Professional Association for Transgender Health (WPATH) released a guideline with numerous recommendations for working with transgender people. Among these, the need to qualify healthcare services stands out, ensuring access, support and comprehensive care for transgender people. In Brazil, progress has already been made on this recommendation, with emphasis on the Brazilian Health System (SUS - *Sistema Único de Saúde*) public policies. However, there is a difficulty in translating these recommendations into clinical practice, with a lack of access to universities^(6)^.

International studies indicate chronic experiences of discrimination, rejection and stigma towards the transgender population in health and community services^(4,5)^. In Brazil, in a study carried out with Brazilian transgender adolescents, in the 27 federative units, the precariousness of healthcare services for this population was observed, which, when accessing them, experiences low professional qualifications and is subjected to the logic of referral and counter-referral, with long waiting lines and few specialized service units, which have an impact on the pilgrimage to services and their suffering^(9)^. In order to change this scenario, it is necessary to recognize the possible barriers to access and welcoming and what can be done to overcome them, and this study has a look at this research object.

The latest WPATH guideline recommends scientific empowerment on transsexuality, with advances in studies that work to reduce stigma, facilitate access to healthcare, respect gender diversity and give new meaning to pathologization of gender identity and expression^(6)^. Added to this is a scoping review that identified 20 research priorities for the area of children and adolescents to be developed between 2020-2029; among these, there is the need to qualify healthcare services and advance access^(10)^. Thus, integrating the recommendations and the indicated priority, the following research question emerged: what are the barriers and facilitating strategies for access and welcoming transgender children and adolescents in the Healthcare Networks (RAS - *Redes de Atenção à Saúde*) indicated by scientific literature?

## OBJECTIVES

To identify barriers and strategies that facilitate access and welcoming transgender children and adolescents in the RAS.

## METHODS

This is an integrative literature review. To this end, five stages were followed: research question identification; eligibility criteria and literature search establishment; definition of the information to be extracted; assessment of included studies; and interpretation/presentation of results^(11)^.

In order to guide this review, we started from the following definitions of key concepts: 1) Barriers were defined as any impasse or obstacle to access and welcoming children and adolescents, and, conversely, facilitators were defined as strategies that make it possible to modify the context and improve care; 2) Access as the entrance to healthcare services; 3) Welcoming as the recognition of the search for healthcare services as legitimate and unique, with qualified action, through different care technologies; and 4) RAS as different healthcare services, at different levels, which work together, with cooperative and interdependent actions, which allow for the provision of continuous and comprehensive care, such as primary care, outpatient services and hospital units^(12,13,14)^. The research question, mentioned above in the introduction section, was constituted by key concepts and the mnemonic PCC, in which: P (population): transgender children and adolescents; C (concept): barriers and facilitating strategies; and C (context): access and welcoming in the RAS, indicated by scientific literature.

Qualitative or quantitative articles, available in full, that indicated barriers to access or welcoming in the RAS and/or strategies to overcome them, for children and adolescents, considering the age group between 6 and 19 years old, published between January 2013 and April 2023, in Portuguese, English or Spanish, were included. Opinion articles, expert consensus, review protocols, abstracts, editorials and theses/dissertations were excluded. It is worth reiterating that the time frame of the search identified studies from the last ten years and the age group above six years, since, at the end of early childhood, in the typical development process, children are already aware of the definition of their gender, with the beginning of searches for healthcare services after identification of gender incongruence^(5)^.

For literature search, the PubMed, EMBASE, Web of Science, CINAHL, Scopus, APA PsycInfo, LILACS and SciELO databases were chosen. Search strategies were formulated for each database with the help of a librarian specialized in integrative review, using descriptors and the Boolean operators AND and/or OR. The search was carried out on May 5, 2023. The descriptors used in the search strategy were based on the research question such as: P: Transexual; Transgender; Gender Diversity; Sexual and Gender Minorities; C: Barriers to Access of Health Services; C: Heath Personnel; Healthcare Workers; Healthcare Personnel; Healthcare Worker; Healthcare Provider; Nursing Care; Comprehensive Health Care. It should be noted that, initially, descriptors related to children and adolescents were used, but the search was reduced to less than 100 articles, which would limit the study; similarly, when using the descriptors of the services that make up the RAS, the same occurred. Thus, it was decided to remove these descriptors, conducting a broader search and using age and professionals working in the network as search filters.

The following stages were followed in data collection: reading titles and abstracts; reading full-text articles; searching for evidence based on articles’ references; and data collection. For systematic collection, an instrument was formulated by the authors, which contained variables related to manuscript characterization, the barriers and facilitators indicated. Data extraction was performed by a pair of researchers, with a third reviewer in case of any discrepancies. To assist in the extraction, EndNote® and Rayyan® were used. The collected data were categorized and entered into a Microsoft Excel® spreadsheet.

To assess the level of evidence, the JBI classification was used, in which the levels are separated into: level I: systematic review or meta-analysis; level II: randomized controlled clinical trial; level III: controlled clinical trial without randomization/quasi-experimental studies; level IV: well-designed cohort or case-control studies; level V: systematic review of qualitative and descriptive studies; level VI: descriptive or qualitative studies; and level VII: authority opinion or expert opinion report. Levels are classified as strong (I and II), moderate (III to V) and weak (VI to VII)^(15)^.

To help interpret the results, the following documents were used: Statute of Children and Adolescents (ECA - *Estatuto da Criança e do Adolescente*)^(16)^; Patients’ Bill of Rights^(17)^; Brazilian National Guidelines for Comprehensive Care and Health of Adolescents and Young People in Health Promotion, Protection and Recovery^(18)^; Brazilian National Policy for Comprehensive Health of Lesbians, Gays, Bisexuals, Transvestites and Transsexuals (LGBTQAI+)^(12)^; and Ordinance on the Transsexualization Process^(19)^.

Ethical review and approval were waived for this study, as it was a literature review, in addition to the Informed Consent Form.

## RESULTS

**Figure 1:**
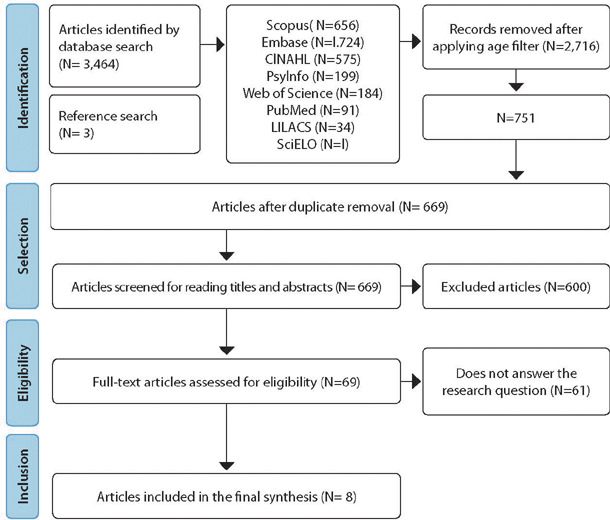
Flowchart of database searches adapted from the Preferred Reporting Items for Systematic Reviews and Meta-Analyses Checklist (PRISMA), São Paulo, São Paulo, Brazil, 2023

**Figure 2:**
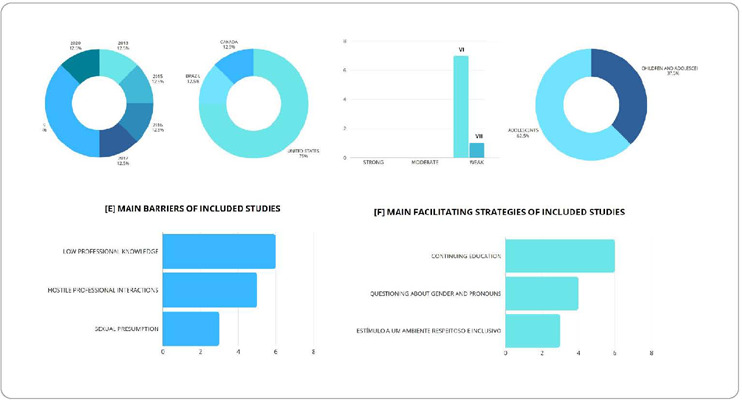
[A] Year of publication; [B] Country of study development; [C] Assessment of the level of evidence; [D] Age group; [E] Main barriers of included studies; and [F] Main facilitating strategies of included studies (N=08), São Paulo, São Paulo, Brazil, 2023

Eight studies were included. Figure 1 shows the search flowchart used in this study, and Chart 1 shows manuscript characterization. These were published between 2013 and 2020 (Figure 2A), with a predominance of studies conducted in the United States (Figure 2B), with a qualitative design, level of evidence VI (Figure 2C), and three were conducted with transgender children and adolescents and five with adolescents (Figure 2D). The main barriers identified were low professional knowledge, hostile professional interactions and sexual presumption (Figure 2E). Strategies included questioning gender identity from the moment of admission to the RAS and healthcare professionals’ continuing education (Figure 2F). The other barriers and strategies are seen in Chart 2.

**Chart 1:** Characterization of included studies (N=08), São Paulo, São Paulo, Brazil. 2023

Author, year and country	Objective	Design and level of evidence	Results/conclusion
American Academy of Pediatrics, 2013, United States^(20)^	Discuss strategies for welcoming transgender children and adolescents in medical offices.	Expert report; Level VII	Experts point to sexual presumption, lack of gender identification, and an unwelcoming environment with structural homophobia as barriers. The manuscript discusses mainly two strategies: approaching transsexuality in a welcoming manner and continuing education for healthcare professionals.
Vance SRJ *et al*.; 2015; United States^(21)^	Explore clinical experiences, comfort, professional confidence, and barriers to providing care to transgender youth.	Qualitative study; Level VI	Professionals reported as barriers low knowledge on the subject, interactions marked by judgments and prejudices linked mainly to the low frequency of care for transgender youth, and lack of interconnection in the RAS. The strategies were training, since graduation, which continues in professionals’ work.
Gridley SJ *et al*.; 2016; United States^(22)^	Understand the barriers that transgender youth and their caregivers face in accessing transgender healthcare.	Qualitative study; Level VI	Young people and their caregivers reported barriers such as the use of registered names, outdated and offensive language used by professionals, hostile interactions, reduced clinical complaints and overvaluation of gender, low professional knowledge, lack of protocols, interconnection between the RAS and low health insurance coverage. The strategies used were questioning gender and pronouns, continuing education, formulation of clinical protocols and respectful and inclusive environments.
Clark BA *et al*.; 2017; Canada^(23)^	Analyze the issues of access to primary care among transgender adolescents and youth.	Qualitative study; Level VI	Young people indicated that the lack of a welcoming space was a barrier to accessing healthcare services, mainly associated with previous negative experiences, limited healthcare service coverage and the fear that communications would not be confidential. The strategies indicate the future possibility of teleconsultations.
Rider GN *et al*.; 2019; United States^(24)^	Analyze the experiences and attitudes of healthcare professionals about working with transgender youth.	Qualitative study; Level VI	Professionals indicated as barriers fear, mainly, in questioning the social name and pronouns, their low knowledge, hostile interactions, which visualize the performance by colleagues, and lack of interconnection between the RAS. The strategies refer to the desire for professional training, with continuing education offered by healthcare services.
Eisenberg ME *et al*.; 2019; United States^(8)^	Describe transgender adolescents’ experiences, concerns, and needs in healthcare settings.	Qualitative study; Level VI	Low professional knowledge was identified as a barrier. As strategies, two main topics were identified in the reports, such as questioning about gender and pronouns, and healthcare professionals’ continuing education. Furthermore, the manuscript indicates the need to focus on health complaints and promote a respectful and inclusive environment.
Acosta W *et al*.; 2019; United States^(25)^	Understand transgender adolescents’ experience in healthcare services.	Qualitative study; Level VI	Adolescents identified as barriers presumption of sexuality, lack of gender identification, repeated use of registered name, low professional knowledge that leads to hostile interactions. Strategies included questioning gender and pronouns in case of unconscious use, apologizing, making an effort to respect, offering space to speak, in a respectful and inclusive environment, and continuing education.
Pontes JC *et al*.; 2020; Brazil^(26)^	Describe and discuss the meanings and concepts attributed by a group of healthcare professionals to the categories of trans “children” and “adolescents” and their relationship with the care practices carried out.	Qualitative study; Level VI	A reduction in transsexuality to transitory stages was observed, with hostile interactions. There is a reduced number of professionals in the multidisciplinary team, and those present tend to downplay complaints and overvalue gender, indicating an unwelcoming environment, with structural homophobia. Strategies were questioning gender identity and the use of pronouns, promoting a space for speech, with a focus on complaints.

**Chart 2:** Strategies to facilitate access and welcoming by transgender children and adolescents to the Healthcare Network, São Paulo, São Paulo, Brazil, 2023

BARRIERS	STRATEGIES
• Professional presumption of transgender children’s and adolescents’ sexuality, with reductionist treatments^(20,24,25)^; • Absence of identification of gender identity with social name and/or pronouns, such as in medical records, identification bracelets and medication administration documentation^(20,25)^; • Unconscious or conscious use of children’s and adolescents’ registered name, often repetitively, even after corrections by patients and/or their family ^(22,25)^; • Professionals are afraid of asking patients about their social name and/or pronouns and making a mistake that could cause offense^(24)^; • Professionals use outdated and offensive language^(18)^; • Lack of a sense of a welcoming space for gender-related health demands, with fear that therapeutic communications would not be confidential^(23)^; • Low professional knowledge, which results in unresponsive care, marked by stigmas and prejudices, with beliefs, personal values and common sense perceptions in the care of both children and adolescents and their families^(8,23,24,25)^; • Hostile interactions between professionals, with minimization of gender identity and association of transsexuality with a transitory stage of human development and/or a pathological view of childhood^(22,24,25,26)^; • Absence of professionals in the multidisciplinary team who could provide welcoming care, such as mental healthcare professionals and social workers^(26)^; • Absence of institutional protocols for welcoming and care of transsexual children and adolescents, with dependence on professional knowledge and judgment for quality care^(22)^; • Reduction in health complaints among children and adolescents, which led them to seek healthcare services for transsexuality, with a lack of interest in the demand for care and gender overvaluation^(22,26)^; • Difficulty in accessing the RAS, due to the lack of specialized services in the region of residence or due to distance, or the perception that the RAS is not available to meet their demands^(23)^; • Lack of interconnection between the RAS, especially specialized and non-specialized centers for the care of transgender children and adolescents, with pilgrimages in search of shelter, especially in cases where care is related to transsexuality^(21,22)^; • Limited health insurance coverage for healthcare related to transsexuality, with the absence of a service that provides care free of charge^(22,23)^; • Unwelcoming healthcare service environment, with structural homophobia^(20,26)^.	• Ask children and adolescents, from the moment they are admitted to the healthcare service, about their gender and the pronouns to be used, in a simple and respectful manner, such as: what are your pronouns? How would you like to be called? The answer should be identified in the medical record, provided that patients and family allow it so that it can be disseminated in the approaches by all professionals in care^(8,20,21,22)^; • In the event of unconscious use of the incorrect name and/or pronoun, it is recommended that professionals not be shy about apologizing to children and adolescents, and call them as they request^(25)^; • Offer a space to speak to transgender children and adolescents, in a respectful manner, with active listening, without judgments and reprisals, acting with empathy, by all professionals who provide assistance, throughout the RAS^(25,26)^; • Training on transsexuality and care for the LGBTQIA+ population from professional graduation onwards^(21,24)^; • Continuing education, with constant training and updates, for all those who communicate with transgender children and adolescents in the RAS. If possible, it is recommended that training be carried out by a transgender person^(8,20,21,24,25)^; • Efforts by healthcare professionals to respect children, adolescents and their families, regardless of gender identity^(25)^; • Development of clear, evidence-based protocols, with an age-appropriate care plan, focusing on children, adolescents and their families^(22)^; • Focus on health complaints brought by children, adolescents and families instead of gender issues, which should be addressed as a way of providing responsive and respectful care, and focused on when the complaint brought is relevant^(8,26)^; • Telecare as a strategy for welcoming transgender children and adolescents with health demands, regardless of where they live, without the need for transportation^(23)^; • Encouraging a respectful and inclusive environment. Environmental adaptations, with posters indicating that children and adolescents are welcome in healthcare services (“This is a neutral place” or “This is a safe place”; use of LGBTQIA+ flag), using pendants on professionals’ clothing that refer to the population. In the long term, construction of neutral bathrooms and beds^(8,22,25)^.

## DISCUSSION

ECA’s seventh article indicates that children and adolescents have the right to protection, life and health, through the implementation of public policies, allowing a healthy and harmonious development in dignified conditions of existence^(16)^. Despite the progress of scientific literature regarding public policies for assistance to the LGBTQIA+ population, a high number of barriers identified in the literature for fulfilling this right for LGBTQIA+ children and adolescents is still observed. These barriers are associated with knowledge, professional conduct and the organization of the RAS and its apparatus. Much progress is still needed, and this study is a starting point for improving practice.

In Brazil, the LGBTQIA+ policy includes, in one of its guidelines, the need to eliminate discrimination against individuals seeking RAS services^(12)^, and, as seen in Chart 1, barriers directly impact this process, and one of them is sexuality presumption. Studies indicate that professionals tend to identify children and adolescents as heterosexual and, even if they make corrections regarding the error in gender and pronouns, professionals tend to continue repeating^(8,21,22,25)^. This misunderstanding may be a reflection of the historical construction of gender issues, contributing to children’s and adolescents’ lack of trust in professionals, with discomfort in an environment that promotes reductionist treatments^(8,22)^.

Furthermore, structurally, healthcare services have not organized themselves to include children’s and adolescents’ social name in their medical records, leading to the recurrent use of their registered name, which contributes to the feeling of the absence of a welcoming space for health demands related to gender^(20,22,25)^.

As a strategy to change this context, studies have reiterated the importance of asking about the gender and pronouns of children or adolescents who will be treated. This question tends to promote acceptance, care, and respect, in addition to increasing patient comfort in the RAS. Transgender people are not born at 18 years of age; they recognize their gender identity in childhood, by adopting symbolic elements socially constructed for boys and girls. Furthermore, by routinely asking about gender and pronouns, professionals can contribute to the fight against the stigma that this population suffers, in addition to normalizing questions on the subject^(8,20,22,25)^.

This questioning can be initiated upon welcoming at RAS services^(8,22,25)^. For instance, when children or adolescents arrive at a healthcare service, initially, when opening the care form, they should be asked about their gender identity, with the record that can be used by all healthcare professionals who will care for transgender children and adolescents. However, even if screening is carried out by a higher level professional, often a nurse^(27)^, there are still barriers.

When highlighting the role of nurses in a Brazilian qualitative study with 20 mothers and two fathers of transgender adolescents, we observed that this professional was mentioned by only one participant, reflecting the gap in access to care provided by professionals and the lack of understanding in civil society about the potential of the profession^(9)^. Furthermore, even if the social name is indicated somewhere in the medical record, professionals may use the registered name by mistake. In this context, it is recommended that professionals do not hesitate to apologize to children and adolescents, calling them as requested^(25)^. All these strategies allow for promoting a space of respectful care for transgender children and adolescents^(25,26)^.

Another aspect is that children and adolescents indicate that only active listening without judgment, with questions such as “What do you need?”, is basically the most important aspect of care, as there is no guide. Despite the existence of guidelines and policies, individuality continues to be the starting point for respectful care that guarantees the rights of children and adolescents^(8,12)^.

Thus, even with the strategies, care can still be marked by stigma, with low professional knowledge, which was the most frequent barrier in the studies^(8,23,24,25)^, added to personal beliefs and values, which reproduce outdated perceptions and common sense, and one of them is the reduction of transsexuality to a transitory phase^(22,24,25,26)^. This perception is a reflection of a historical construction that needs to be reinterpreted. There is the use of biomedical criteria and sociocultural conceptions of gender that reveal impasses and controversies, with the vision of a deviation from normality^(26,28)^. However, reflection is encouraged: what is normal? Furthermore, transgender children and adolescents tend to suffer high rates of internalizing psychopathology, which is perpetuated by spaces that should help to change this context^(25)^.

ECA states, in its fifth article, that no child or adolescent should suffer any form of discrimination^(16)^, and the Patients’ Bill of Rights states that every citizen has the right to humane, welcoming care free from any form of discrimination^(17)^. This study shows that there is still a long way to go to achieve these aspects.

Although included studies did not work with the families of young people, it is important to reflect on this context. In clinical practice, it is observed that many families disseminate reductionist views about transsexuality, and the strategies indicated should also be designed for this audience. Another aspect is that the hostile care provided by professionals directly impacts families, accentuating their suffering. In pediatric nursing care, Family-Centered Care is a model to be followed. In this model, the family is seen as an essential partner in care, and its implementation in RAS services can be a strategy for access and welcoming transgender children and adolescents, considering that the service can be seen by their families as a source of care^(29)^.

Studies have indicated that vital strategies to address the main barrier are training of healthcare professionals and continuing education^(8,20,21,22,24,25)^. It is estimated that, on average, five hours of medical training courses are dedicated to LGBTQIA+ health, without a specific context for transsexuality, requiring professionals to independently seek out ways to improve their skills. This estimate is even lower in nursing courses, which last between one and two hours^(30)^.

In continuing education, it is recommended to focus on teaching basic gender relations, diversity, experiences and strategies for dignified care^(8)^. It is worth noting that these trainings should not be taken on a one-off basis, without subsequent updating; for the strategy to be truly effective, consistency is recommended. Another aspect is that education should not be restricted to healthcare professionals, since the RAS is made up of several professions that must also be improved^(20)^. In this context, the formulation of institutional protocols^(22)^ can be a strategy to promote humanized and sensitive care for transgender children and adolescents.

In international studies, the indication of paid and limited coverage of health insurance for care related to transsexuality was observed as a barrier to access^(22,23)^. In Brazil, public policies guarantee universal and comprehensive access to RAS for LG-BTQIA+ children and adolescents in the SUS free of charge^(17,18,19,22)^, positive aspect that should be visible, demonstrating the potential of a public health network. Here, other problems may be more frequent, such as RAS interconnection and the distance from qualified healthcare services.

The lack of interconnection between the RAS, especially between primary care and specialized care, is a challenge for the Brazilian health network that goes beyond transgender children’s and adolescents’ health. This context leads to a pilgrimage between services, with a lack of longitudinal care^(21,22)^. Collective efforts need to be made to change this scenario, and the strategies listed in Chart 1 can be integrated into implementation studies to ensure this coordination.

There is also the difficulty of access, whether due to place of residence or distance. One strategy recommended in this context is telecare, which has gained greater visibility after the COVID-19 pandemic^(23)^. It is worth noting that, even in remote care, all the strategies mentioned in Chart 1 must be integrated for welcoming care.

When portraying this access, it is necessary to reflect that, in addition to transsexuality, as children grow up and experience adolescence, healthcare services are no longer accessed frequently, with the reduction in the number of childcare consultations in primary care, the gateway to the network^(19)^. Professionals’ focus is on early childhood instead of subsequent stages and the process of illness so that adolescents are not seen by public policies. In the health system, actions are focused on harm reduction, sexual education with a focus on preventing sexually transmitted infections and pregnancy, with a reduction in assistance for reproductive functions, leaving aside all the uniqueness of the phase^(3)^. Thus, intrinsically, RAS ceases to be a potential in children’s and adolescents’ lives, having repercussions throughout adulthood^(3,9)^. This may be more frequent in cases of transsexuality.

The Patients’ Bill of Rights^(17)^ states that every citizen has the right to adequate and effective treatment for their care needs. However, in this study, it was observed that transgender children’s and adolescents’ complaints tend to be focused on gender, regardless of the demand^(22,26)^. An illustration of this are transgender children and adolescents who seek services due to respiratory problems, but the health staff, despite treating this complaint, focuses all care on the fact that they are transgender, which can increase their exclusion from healthcare services, especially if this view of transsexuality is marked by stigma and pathologization of gender identity.

As an illustration, in an included study, a 16-year-old adolescent reported that: “*I am very sick, but I don’t want to have to deal with all the hate in the doctor’s office or all the discrimination against me*”. The adolescent emphasizes not going to the RAS because feels anxious that will not be accepted there: “*It is hard to go to the doctor if you are hated there*”^(8)^, reiterating the need for acceptance. It is worth noting that transsexuality should be the leading actor of care when the search for the service is due to this and, in other cases, care should occur in a way that respects gender identity, but acts on the underlying complaint that led children or adolescents to healthcare services^(23)^.

In the end, the environment of services and structural homophobia were also identified barriers^(20,26)^. Chart 1 presents strategies for changing the environment, but it is important to emphasize that this is not valid, in isolation, if the care provided by healthcare professionals is not welcoming. When promoting a physical environment prepared to receive transgender children and adolescents, it is necessary for professionals to be trained to assist the population.

The access and welcoming strategies presented in this study demonstrate that transgender children and adolescents do not want to be treated differently or in a special manner, but rather want their care to be the same as that of any other patient, without the need to hide who they are. All of the welcoming measures presented here can be put into practice, as long as a healthcare service is willing. Staff training, protocol development and frequent auditing should be implemented in all healthcare services, from primary care to tertiary services, public or private. These measures would demonstrate that the staff of these healthcare services are respectful, welcoming and provide a safe environment for any type of treatment^(12)^. However, it is known that these strategies have intrinsic limitations, such as isolated training without consistency. Thus, it is necessary to reflect on an intersection between the numerous strategies indicated in this article and the context of action, in order to increase the chance of success in clinical practice.

### Study limitations

This study has the limitation of centralizing qualitative studies, which does not allow demonstrating the magnitude of the impacts of the barriers and strategies indicated. However, this aspect does not reduce the potential of the data. In LGBTQIA+ policy, the need for scientific knowledge production is indicated as a guideline^(12)^, with this study being included in this aspect.

### Contributions to health

It is expected that this review will contribute to the reorientation and/or construction of guidelines or public policies that aim to guarantee access and welcoming for transgender children and adolescents in an interconnected and optimized RAS to recognize, validate and welcome their gender identity, with appropriate, safe and inclusive care.

## CONCLUSIONS

This study demonstrated the barriers experienced by transgender children and adolescents in the RAS and the strategies to overcome them. In eight studies, it was observed that the main barriers were low professional knowledge, hostile professional interactions and sexual presumption. The strategies included questioning gender identity, from the time of admission to the RAS, and healthcare professionals’ continuing education. It is expected that, with the demonstration of this context, healthcare professionals will be able to reflect on their practices, in order to minimize impasses and enhance universal and welcoming access for transgender children and adolescents, with the interconnection of the aforementioned strategies in their care, in addition to moving forward with implementation studies that enable the translation of existing public policies into clinical practice, in order to respect the rights of transgender children and adolescents.
