# Supplementary material for: Barriers and facilitating strategies for healthcare access and reception for transgender children and adolescents
Source: Rev Bras Enferm. 2025 Mar 17;78(Suppl 2):e20240266. doi: 10.1590/0034-7167-2024-0266 (PMC11913426; doi:10.1590/0034-7167-2024-0266)
Supplement: Translation

# Translation: Barreiras e estratégias facilitadoras no acesso e acolhimento em saúde de crianças e adolescentes transexuais

**DOI:** 10.1590/0034-7167-2024-0266pt

**Authors:** Juliana Cristina Pereira Silveira, Danton Matheus de Souza, Clarissa de Souza Cardoso, Márcia Aparecida Ferreira de Oliveira

**Affiliations:** I Universidade de São Paulo. São Paulo, São Paulo, Brasil; II Universidade Federal de Pelotas. Pelotas, Rio Grande do Sul, Brasil

**Keywords:** Pessoas Transgênero, Minorias Sexuais e de Gênero, Criança, Adolescente, Barreiras ao Acesso aos Cuidados de Saúde

## Abstract

**Objetivos:**

identificar as barreiras e as estratégias facilitadoras de acesso e acolhimento de crianças e adolescentes transexuais na Rede de Atenção à Saúde.

**Métodos:**

revisão integrativa da literatura, com artigos, disponíveis na íntegra, com crianças e adolescentes entre 6 e 19 anos incompletos, publicados entre janeiro de 2013 e abril de 2023, nos idiomas português, inglês ou espanhol. O nível de evidência foi avaliado com a classificação do JBI.

**Resultados:**

as principais barreiras foram baixo conhecimento profissional, interações profissionais hostis e presunção sexual. As estratégias facilitadoras foram educação continuada, questionamento sobre gênero e pronome, e estímulo a um ambiente respeitoso e acolhedor.

**Conclusões:**

este estudo indica inúmeras barreiras para o acesso e acolhimento de crianças e adolescentes transexuais e estratégias passíveis de transformar esse contexto, visando caminhar em busca de um atendimento integral que reconheça, valide e acolha a identidade de gênero com cuidados apropriados, seguros e inclusivos.

## INTRODUÇÃO

Transexualidade refere-se a uma pessoa que não identifica o seu gênero com o sexo que lhe foi designado ao nascimento. O termo “trans” é amplamente utilizado para abranger essa população, que reivindica socialmente seu reconhecimento^(1)^. Assim, mulheres trans são aquelas que nasceram com sexo masculino, mas que se identificam com o gênero feminino; homens trans são aqueles nascidos com sexo feminino, porém se identificam com o gênero masculino; e pessoas do gênero não binário (GNB) são aquelas que sentem que sua identidade está fora ou entre as identidades femininas e masculinas, identificando-se com ambos ou nenhuma^(1,2)^.

Esse processo de identificação surge na etapa infantojuvenil, de forma gradual, com início na primeira infância. Ao final desta, a criança já tem consciência da definição do seu gênero. Porém, a identificação de gênero pode não ser referida devido à representação social do cisgênero e estigmatização da transexualidade, podendo ser referida na adolescência^(3)^. Cabe salientar que o reconhecimento da existência de crianças e adolescentes transexuais significa a defesa das suas necessidades e desejos como seres humanos e sujeitos de direitos, a partir de uma reinvindicação da expressividade de gênero diferente da cisgeneridade^(3)^. Nos últimos anos, observa-se um aumento de estudos relacionados à população transexual, porém com enfoque em adultos. Poucas investigações atuaram com crianças e adolescentes, sendo que as existentes focalizaram nas repercussões da transfobia em sua saúde mental^(4,5)^.

Estima-se uma proporção mundial de indivíduos que se identificam como transgênero entre 0,1% e 2% da população geral^(6)^. No Brasil, estudo que avaliou a proporção da diversidade de gênero mostrou que, de 5.930 pessoas acima de 18 anos, 1,9% possuem diversidade de gênero, sendo 0,7% transexuais e 1,2% GNB. Ainda, estima-se que cerca de 1.090.200 adultos possam se identificar como transexuais e, 1.880.200, como GNB, totalizando, assim, quase 3.000.000 de pessoas possuindo diversidade de gênero^(1)^. Na etapa infantojuvenil, a literatura ainda diverge, com uma média entre 0,1% e 1,3%^(6,7)^. Porém, esses estudos podem não ser precisos na proporção de indivíduos transexuais, principalmente com crianças e adolescentes, uma vez que a identificação é mascarada em um contexto de estigmas e invisibilidade da transexualidade infantojuvenil^(1,3,8)^.

Recentemente, de forma a avançar na temática, a *World Professional Association for Transgender Health* (WPATH) divulgou um *guideline* com inúmeras recomendações para a atuação com pessoas transexuais. Entre essas, destaca-se a necessidade de qualificação dos serviços de saúde, garantindo acesso, acolhimento e assistência integral à pessoa transexual. No Brasil, avanço nessa recomendação já foi feito, com destaque nas políticas públicas do Sistema Único de Saúde (SUS). Todavia, observa-se uma dificuldade de tradução dessas recomendações à prática clínica, com uma universidade de acesso falha^(6)^.

Estudos internacionais indicam experiências crônicas de discriminação, rejeição e estigma à população transexual em serviços de saúde e comunitários^(4,5)^. No Brasil, em investigação realizada com adolescentes transexuais brasileiros, nas 27 unidades federativas, observou-se a precariedade dos serviços de saúde a essa população que, quando o acessa, vivencia a baixa qualificação profissional e é submetida à lógica da referência e contrarreferência, com longas filas de espera e poucas unidades de serviço especializadas, que repercutem na peregrinação nos serviços e em seu sofrimento^(9)^. De forma a alterar esse cenário, é necessário reconhecer as eventuais barreiras de acesso e acolhimento e o que pode ser feito para superá-las, sendo que este estudo possui um olhar a este objeto de pesquisa.

O último *guideline* do WPATH traz como recomendações o empoderamento científico na temática da transexualidade, com avanço em estudos que atuem na redução do estigma, facilitação do acesso aos cuidados de saúde, respeito à diversidade de gênero e ressignificação da patologização da identidade e expressão de gênero^(6)^. Soma-se a isso revisão de escopo que identificou 20 prioridades de pesquisa para a área infantojuvenil a serem desenvolvidas entre 2020-2029; entre essas, há a necessidade de qualificação dos serviços de saúde e avanço no acesso^(10)^. Assim, integrando as recomendações e a prioridade indicada, emergiu a seguinte pergunta de pesquisa: quais são as barreiras e as estratégias facilitadoras de acesso e acolhimento de crianças e adolescentes transexuais nas Redes de Atenção à Saúde (RAS) indicadas pela literatura científica?

## OBJETIVOS

Identificar as barreiras e as estratégias facilitadoras de acesso e acolhimento de crianças e adolescentes transexuais na RAS.

## MÉTODOS

Trata-se de revisão integrativa da literatura. Para tal, foram percorridas cinco etapas: identificação da pergunta de pesquisa; estabelecimento dos critérios de elegibilidade e busca na literatura; definição das informações a serem extraídas; avaliação dos estudos incluídos; e interpretação/apresentação dos resultados^(11)^.

De forma a guiar esta revisão, partimos das seguintes definições dos conceitos-chave: 1) Barreiras foram definidas como qualquer impasse ou obstáculo para que o acesso e o acolhimento de crianças e adolescentes ocorram, e, de forma contrária, os facilitadores foram definidos como estratégias que possibilitam modificar o contexto e aperfeiçoar a assistência; 2) Acesso como a entrada aos serviços de saúde; 3) Acolhimento como o reconhecimento da busca ao serviço de saúde como legítima e singular, com atuação qualificada, por intermédio de diferentes tecnologias de cuidado; e 4) RAS como diferentes serviços de saúde, de diferentes níveis, que atuam em conjunto, com ações cooperativas e interdependentes, que permitem ofertar atenção contínua e integral, como atenção primária, serviços ambulatoriais e unidades hospitalares^(12,13,14)^. A pergunta de pesquisa, supracitada na sessão da introdução, foi constituída pelos conceitos-chave e pelo mnemônico PCC, em que: P (população): crianças e adolescentes transexuais; C (conceito): barreiras e estratégias facilitadoras; e C (contexto): acesso e acolhimento na RAS, indicados pela literatura científica.

Foram incluídos artigos com delineamento qualitativo ou quantitativo, disponíveis na íntegra, que indicassem barreiras de acesso ou acolhimento na RAS e/ou estratégias para superá-las, por crianças e adolescentes, considerando a faixa etária entre 6 e 19 anos incompletos, publicados entre janeiro de 2013 e abril de 2023, nos idiomas português, inglês ou espanhol. Foram excluídos artigos de opinião, consensos de especialistas, protocolos de revisão, resumos, editoriais e teses/dissertações. Reitera-se que o recorte temporal de busca ocorreu de forma a capturar os estudos dos últimos dez anos e a faixa etária acima de seis anos, uma vez que, ao final da primeira infância, no processo típico do desenvolvimento, a criança já possui consciência da definição do seu gênero, com o início das buscas aos serviços de saúde após identificação da incongruência de gênero^(5)^.

Para a busca na literatura, foram escolhidas as bases de dados PubMed, EMBASE, *Web of Science*, CINAHL, Scopus, APA PsycInfo, LILACS e SciELO. Foram formuladas estratégias de busca para cada base de dados com auxílio de uma bibliotecária especialista em revisão integrativa, com o uso de descritores e os operadores booleanos *AND* e/ou *OR*. A busca foi realizada no dia 05 de maio de 2023. Os descritores utilizados na estratégia de busca foram baseados na pergunta de pesquisa como: P: *Transexual; Transgender; Gender Diversity; Sexual and Gender Minorities;* C: *Barriers to Access of Health Services;* C: *Heath Personnel; Healthcare Workers; Healthcare Personnel; Healthcare Worker; Healthcare Provider; Nursing Care; Comprehensive Health Care*. Reitera-se que, inicialmente, utilizaram-se descritores relacionados à criança e ao adolescente, porém a busca foi reduzida a menos de 100 artigos, o que limitaria o estudo; de forma semelhante, ao fazer uso dos descritores dos serviços que compõem a RAS, o mesmo ocorreu. Assim, optou-se por retirar esses descritores, fazendo uma busca mais ampla e colocando a idade e os profissionais atuantes na rede como filtro de pesquisa.

Na coleta de dados, seguiram-se as seguintes etapas: leitura de títulos e resumos; leitura do artigo na íntegra; busca de evidências com base nas referências dos artigos; e coleta de dados. Para uma coleta sistematizada, foi formulado um instrumento, pelos autores, que continha variáveis referentes à caracterização do manuscrito, às barreiras e aos facilitadores indicados. A extração dos dados foi realizada por uma dupla de pesquisadores, com um terceiro revisor em caso de alguma divergência. Para auxiliar na extração, utilizaram-se o programa *EndNote*® e o *software Rayyan*®. Os dados coletados foram categorizados e digitados em uma planilha do *Microsoft Excel*®.

Para a avaliação do nível de evidência, utilizou-se a classificação do JBI, em que há a separação de níveis em: nível I: revisão sistemática ou metanálise; nível II: ensaio clínico controlado randomizado; nível III: ensaio clínico controlado sem randomização/estudos quase-experimentais; nível IV: estudos de coorte ou caso-controle bem delineados; nível V: revisão sistemática de estudos qualitativos e descritivos; nível VI: estudos descritivos ou

qualitativos; e nível VII: opinião de autoridades ou relato de especialistas. Os níveis são classificados em forte (I e II), moderado (III a V) e fraco (VI a VII)^(15)^.

Para auxílio na interpretação dos resultados, utilizaram-se os seguintes documentos: Estatuto da Criança e do Adolescente (ECA)^(16)^; Carta de Direito dos Usuários de Saúde^(17)^; Diretrizes Nacionais para a Atenção Integral e Saúde de Adolescentes e Jovens na Promoção, Proteção e Recuperação em Saúde^(18)^; Política Nacional de Saúde Integral de Lésbicas, Gays, Bissexuais, Travestis e Transexuais (LGBTQAI+)^(12)^; e Portaria do Processo Transexualizador^(19)^.

A revisão ética e a aprovação foram dispensadas para este estudo, devido a ser uma revisão da literatura, além do Termo de Consentimento Livre e Esclarecido.

## RESULTADOS

Foram incluídos oito estudos. A Figura 1 traz o fluxograma de busca utilizado neste estudo, e o Quadro 1, a caracterização dos manuscritos. Esses foram publicados entre 2013 e 2020 (Figura 2A), prevalecendo os estudos conduzidos nos Estados Unidos (Figura 2B), com delineamento qualitativo, de nível de evidência VI (Figura 2C), e três foram conduzidos com crianças com adolescentes transexuais e cinco com adolescentes (Figura 2D). As principais barreiras identificadas foram baixo conhecimento profissional, interações profissionais hostis e presunção sexual (Figura 2E). As estratégias foram o questionamento da identidade de gênero desde a admissão na RAS e a educação continuada de profissionais de saúde (Figura 2F). As demais barreiras e estratégias são vistas no Quadro 2.

**Figura 1:** Fluxograma das buscas nas bases de dados adaptados do *Preferred Reporting Items for Systematic Reviews and Meta-Analyses Checklist* (PRISMA), São Paulo, São Paulo, Brasil, 2023

**Quadro 1:** Caracterização dos estudos incluídos (N=08), São Paulo, São Paulo, Brasil. 2023

Autor, ano e país	Objetivo	Delineamento e nível de evidência	Resultados/conclusão
*American Academy of Pediatrics,* 2013, Estados Unidos^(20)^	Discorrer sobre estratégias de acolhimento a crianças e adolescentes transexuais nos consultórios médicos.	Relatório de especialista; Nível VII	Os especialistas indicam como barreiras presunção sexual, ausência da identificação de gênero, ambiência pouco acolhedora com homofobia estrutural. O manuscrito discorre, principalmente, sobre duas estratégias: abordagem da transexualidade de forma acolhedora e educação continuada de profissionais de saúde.
Vance SRJ *et al*.; 2015; Estados Unidos^(21)^	Explorar as experiências clínicas, o conforto, a confiança dos profissionais e as barreiras para prestar cuidados a jovens transgêneros.	Estudo qualitativo; Nível VI	Os profissionais relataram como barreiras baixo conhecimento sobre a temática, interações marcadas por julgamentos e preconceitos ligados, principalmente, à baixa frequência de atendimentos aos jovens transexuais, e ausência entre interligação na RAS. As estratégias foram formação, desde a graduação, que se segue na atuação do profissional.
Gridley SJ *et al*.; 2016; Estados Unidos^(22)^	Compreender as barreiras que os jovens transexuais e seus cuidadores enfrentam no acesso a cuidados de saúde transexuais.	Estudo qualitativo; Nível VI	Os jovens e seus cuidados referiram como barreira uso do nome registrado, linguagem desatualizada e ofensiva dos profissionais, interações hostis, redução das queixas clínicas e supervalorização do gênero, baixo conhecimento profissional, ausência de protocolos, interligação entre a RAS e baixa cobertura do seguro de saúde. As estratégias foram questionamento do gênero e pronome, educação continuada, formulação de protocolos clínicos e ambientes respeitosos e inclusivos.
Clark BA *et al*.; 2017; Canadá^(23)^	Analisar as questões do acesso aos cuidados primários entre adolescentes e jovens transgêneros.	Estudo qualitativo; Nível VI	Os jovens indicaram como barreira para o acesso ao serviço de saúde ausência de espaço acolhedor, associado, principalmente, a experiências anteriores negativas, à cobertura limitada de serviços de saúde e ao medo de que as comunicações não fossem confidenciais. As estratégias indicam a possibilidade futura de teleatendimentos.
Rider GN *et al*.; 2019; Estados Unidos^(24)^	Analisar as experiências e atitudes dos profissionais de saúde sobre trabalhar com jovens transexuais.	Estudo qualitativo; Nível VI	Os profissionais indicaram como barreiras medo, principalmente, em questionar o nome social e pronomes, seu baixo conhecimento, interações hostis, que visualizam a realização por colegas, e ausência da interligação entre a RAS. As estratégias referem-se ao desejo de formação profissional, com educação continuada ofertada pelos serviços de saúde.
Eisenberg ME *et al*.; 2019; Estados Unidos^(8)^	Descrever as experiências, preocupações e necessidades dos adolescentes transexuais nos serviços de saúde.	Estudo qualitativo; Nível VI	Identificou-se como barreira baixo conhecimento profissional. Como estratégias, dois temas principais foram identificados nos relatos, como questionamento sobre o gênero e pronome, e educação continuada de profissionais de saúde. Ainda, o manuscrito indica a necessidade do enfoque nas queixas de saúde e promoção de um ambiente respeitoso e inclusivo.
Acosta W *et al*.; 2019; Estados Unidos^(25)^	Compreender a experiência de adolescentes transgêneros nos serviços de saúde.	Estudo qualitativo; Nível VI	Os adolescentes identificaram como barreiras presunção da sexualidade, ausência de identificação de gênero, com uso repetido do nome registrado, baixo conhecimento profissional que leva a interações hostis. As estratégias foram questionamento sobre gênero e pronome, em caso de uso inconsciente, pedir desculpa, com esforço para respeitar, ofertar espaço de fala, em um ambiente respeitoso e inclusivo, e educação continuada.
Pontes JC *et al*.; 2020; Brasil^(26)^	Descrever e discutir os significados e as concepções atribuídos por um grupo de profissionais de saúde às categorias “crianças” e “adolescentes” trans e sua relação com as práticas de cuidado realizadas.	Estudo qualitativo; Nível VI	Observou-se uma redução da transexualidade a etapas transitórias, com interações hostis. Há um número reduzido de profissionais da equipe multiprofissional, e os presentes tendem a reduzir as queixas e supervalorizar o gênero, indicando a ambiência pouco acolhedora, com a homofobia estrutural. As estratégias foram questionamento da identidade de gênero e uso de pronomes, promoção de um espaço de fala, com enfoque nas queixas.

**Figura 2:** [A] Ano de publicação; [B] País de desenvolvimento do estudo; [C] Avaliação do nível de evidência; [D] Faixa etária; [E] Principais barreiras dos estudos incluídos; e [F] Principais estratégias facilitadoras dos estudos incluídos (N=08), São Paulo, São Paulo, Brasil, 2023

**Quadro 2:** Estratégias facilitadoras para o acesso e acolhimento à Rede de Atenção à Saúde por crianças e adolescentes transexuais, São Paulo, São Paulo, Brasil, 2023

BARREIRAS	ESTRATÉGIAS
• Presunção profissional da sexualidade das crianças e dos adolescentes transexuais, com tratamentos reducionais^(20,24,25)^; • Ausência da identificação da identidade de gênero com o nome social e/ou pronomes, como nos prontuários, nas pulseiras de identificação e na documentação de administração de medicações^(20,25)^; • Uso inconsciente ou consciente do nome registrado da criança e do adolescente, frequentemente, de forma repetitiva, mesmo após correções do paciente e/ou sua família^(22,25)^; • Medo dos profissionais em questionar o nome social e/ou pronome dos pacientes e cometer um equívoco que cause ofensas^(24)^; • Uso de linguagem desatualizada e ofensiva pelos profissionais^(18)^; • Ausência da sensação de um espaço acolhedor para demandas de saúde relacionadas ao gênero, com medo de que as comunicações terapêuticas não fossem confidenciais^(23)^; • Baixo conhecimento profissional, que repercute em um cuidado pouco responsivo, marcado por estigmas e preconceitos, com crenças, valores pessoais e percepções do senso comum no cuidado tanto à criança e ao adolescente quanto à sua família^(8,23,24,25)^; • Interações hostis dos profissionais, com minimização da identidade de gênero e ligação da transexualidade a uma etapa transitória do desenvolvimento humano e/ou visão patológica da infância^(22,24,25,26)^; • Ausência de profissionais na equipe multiprofissional que poderiam prestar um cuidado acolhedor, como os profissionais de saúde mental e os assistentes sociais^(26)^; • Ausência de protocolos institucionais para o acolhimento e cuidado à criança e ao adolescente transexual, com dependência dos conhecimentos e senso profissional para um cuidado de qualidade^(22)^; • Redução da queixa de saúde da criança e do adolescente, que os fizeram buscar o serviço de saúde, para a transexualidade, com falta de interesse na demanda de cuidado e supervalorização do gênero^(22,26)^; • Dificuldade de acessar a RAS, pela ausência de serviços especializados na região de moradia ou pela distância, ou percepção de que a RAS não está disponível para acolher suas demandas^(23)^; • Ausência de interligação entre a RAS, especialmente os centros especializados e não especializados no cuidado à criança e ao adolescente transexual, com peregrinação em busca de acolhimento, principalmente em casos em que o cuidado está relacionado à transexualidade^(21,22)^; • Cobertura limitada do seguro de saúde aos cuidados de saúde relacionados à transexualidade, com ausência de serviço que preste a atenção de forma gratuita^(22,23)^; • Ambiência dos serviços de saúde pouco acolhedoras, com homofobia estrutural^(20,26)^.	• Questionar a criança e o adolescente, desde a admissão no serviço de saúde, sobre o seu gênero e pronome a ser utilizado, de forma simples e respeitosa, como: quais são seus pronomes? Como você gostaria de ser chamado? A resposta deve ser identificada em prontuário, desde que o paciente e família permitam, para que seja disseminado nas abordagens por todos os profissionais no cuidado^(8,20,21,22)^; • Em caso de ocorrência inconsciente do uso do nome e/ou pronome incorreto, recomenda-se que o profissional não se acanhe em pedir desculpas à criança e ao adolescente, e chame-o conforme ele solicita^(25)^; • Ofertar um espaço de fala à criança e ao adolescente transexual, de forma respeitosa, com escuta ativa, sem julgamentos e represálias, atuando com empatia, por todos os profissionais que prestem assistência, em toda a RAS^(25,26)^; • Formação sobre transexualidade e cuidados à população LGBTQIA+ desde a graduação profissional^(21,24)^; • Educação continuada, com treinamentos e atualizações constantes, a todos que se comunicam com a criança e o adolescente transexual na RAS. Se possível, recomenda-se que o treinamento seja realizado por uma pessoa transexual^(8,20,21,24,25)^; • Esforço dos profissionais de saúde em respeitar as crianças, os adolescentes e suas famílias, independentemente da identidade de gênero^(25)^; • Desenvolvimento de protocolos claros e baseados em evidência, com roteiro adequado de cuidado à idade, com foco na criança, no adolescente e em sua família^(22)^; • Focar nas queixas de saúde trazidas pela criança, pelo adolescente e pela família ao invés de questões de gênero, que devem ser abordadas como forma de prestar um cuidado responsivo e respeitoso, e focalizadas quando for relevante a queixa trazida^(8,26)^; • Teleatendimento como estratégia para o acolhimento de crianças e adolescentes transexuais com demandas de saúde, independentemente do local em que vivem, sem a necessidade de locomoção^(23)^; • Estímulo a um ambiente respeitoso e inclusivo. Adaptações ambientais, com cartazes que indiquem que a criança e o adolescente são bem-vindos ao serviço de saúde (“Aqui é um lugar neutro” ou “Aqui é um lugar seguro”; uso da bandeira LGBTQIA+), usando pingentes na roupa dos profissionais que remetam à população. A longo prazo, construção de banheiros e leitos neutros^(8,22,25)^.

## DISCUSSÃO

O sétimo artigo do ECA indica que a criança e o adolescente têm o direito à proteção, à vida e à saúde, mediante a efetivação das políticas públicas, permitindo um desenvolvimento sadio e harmonioso em condições dignas de existência^(16)^. Apesar do avançar da literatura científica diante das políticas públicas para a assistência à população LGBTQIA+, ainda se observa um alto número de barreiras identificadas na literatura para a efetivação desse direito nas crianças e adolescentes LGBTQIA+. Essas barreiras estão associadas ao conhecimento, à conduta profissional e à organização da RAS e dos seus aparatos. Um longo avanço ainda é necessário, sendo este estudo um pontapé inicial para o aperfeiçoamento da prática.

No Brasil, a política de LGBTQIA+ traz, em uma de suas diretrizes, a necessidade da eliminação da discriminação dos indivíduos que buscam os serviços da RAS^(12)^, e, como visto no Quadro 1, barreiras impactam diretamente esse processo, e uma delas é a presunção da sexualidade. Os estudos indicam que os profissionais tendem a identificar as crianças e adolescentes como heterossexuais e, mesmo que essas façam correções sobre o erro do gênero e dos pronomes, o profissional tende a seguir repetindo^(8,21,22,25)^. Esse equívoco pode ser um reflexo da construção histórica das questões de gênero, contribuindo para a falta de confiança das crianças e adolescentes nos profissionais, com desconforto em um ambiente que promove tratamentos reducionistas^(8,22)^.

Ainda, estruturalmente, os serviços de saúde não se organizaram para a inclusão do nome social das crianças e dos adolescentes em seus prontuários, levando ao uso recorrente do nome registrado, que contribui para a sensação da ausência de um espaço acolhedor para as demandas de saúde relacionadas ao gênero^(20,22,25)^.

Como estratégia para modificar esse contexto, os estudos reiteraram a importância de perguntar sobre o gênero e o pronome da criança ou do adolescente que será atendida/atendido. A pergunta tende a promover um acolhimento, cuidado e respeito, além de aumentar o conforto do paciente na RAS. Pessoas transgêneros não nascem com 18 anos; elas reconhecem sua identidade de gênero ainda na infância, ao adotar elementos simbólicos construídos socialmente para meninos e meninas. Além disso, ao perguntar rotineiramente sobre gênero e pronome, os profissionais podem contribuir na luta contra o estigma que essa população sofre, além de normalizar as questões sobre o tema^(8,20,22,25)^.

Esse questionamento pode ser iniciado desde a recepção nos serviços da RAS^(8,22,25)^. A título de ilustração, quando a criança ou o adolescente chegar ao serviço de saúde, inicialmente, na abertura da ficha de atendimento, esse deve ser questionado sobre a sua identidade de gênero, com o registro que poderá ser utilizado por todos os profissionais de saúde que cuidarão da criança e adolescente transexual. Contudo, mesmo que a triagem seja realizada por um profissional de nível superior, frequentemente um(a) enfermeiro(a)^(27)^, ainda há barreiras.

Ao ressaltarmos o papel do enfermeiro em estudo qualitativo, brasileiro, com 20 mães e dois pais de adolescentes transexuais, observou-se a citação desse profissional por apenas uma participante, retratando a lacuna de acesso aos cuidados realizados pelo profissional e a falta de compreensão da sociedade civil sobre as potencialidades da profissão^(9)^. Ainda, mesmo com o nome social indicado em algum local do prontuário, os profissionais podem usar o nome registrado por engano. Nesse contexto, recomenda-se que ele não se acanhe em pedir desculpas à criança e ao adolescente, chamando-o conforme se solicita^(25)^. Todas essas estratégias permitem que seja promovido um espaço de cuidado respeitoso para a criança e o adolescente transexual^(25,26)^.

Outro aspecto é que as crianças e adolescentes indicam que somente a escuta ativa sem julgamentos, com questionamentos como “O que você precisa?”, é basicamente o aspecto mais importante no cuidado, pois não há um guia. Apesar da existência de diretrizes e políticas, a individualidade segue sendo o ponto de partida para um cuidado respeitoso que garanta os direitos das crianças e adolescentes^(8,12)^.

Assim, mesmo com as estratégias, o cuidado ainda pode ser marcado pelo estigma, com o baixo conhecimento profissional, que foi a barreira mais frequente nos estudos^(8,23,24,25)^, somado às crenças e aos valores pessoais, que reproduzem percepções ultrapassadas e do senso comum, e uma delas é a redução da transexualidade a uma fase transitória^(22,24,25,26)^. Essa percepção é reflexo de uma construção histórica que precisa ser ressignificada. Há o uso de critérios biomédicos e concepções socioculturais de gênero que revelam impasses e controvérsias, com a visão de um desvio da normalidade^(26,28)^. Contudo, estimula-se a reflexão: o que é o normal? Ainda, crianças e adolescentes transexuais tendem a sofrer altas taxas de psicopatologia internalizante, que é perpetuada pelos espaços que deveriam auxiliar na modificação desse contexto^(25)^.

O ECA indica, em seu quinto artigo, que nenhuma criança ou adolescente deve sofrer formas de discriminação^(16)^, e a Carta de Direito dos Usuários de Saúde indica que todo cidadão possui o direito ao atendimento humanizado, acolhedor e livre de qualquer forma de discriminação^(17)^. Este estudo demonstra que ainda há um longo caminho a ser seguido para se atingir esses aspectos.

Apesar de os estudos incluídos não terem atuado com as famílias dos jovens, cabe refletir esse contexto. Na prática clínica, observa-se que muitas famílias disseminam visões reducionistas sobre a transexualidade, e as estratégias indicadas devem, também, ser pensadas para esse público. Outro aspecto é que o cuidado hostil prestado pelos profissionais impacta diretamente as famílias, acentuando seu sofrimento. Na assistência de enfermagem pediátrica, o Cuidado Centrado na Família é um modelo a ser seguido. Neste, a família é tida como uma parceira essencial do cuidado, e a sua implementação nos serviços da RAS pode ser uma estratégia para acesso e acolhimento das crianças e dos adolescentes transexuais, considerando que o serviço pode ser visto por seus familiares como uma fonte de cuidado^(29)^.

Os estudos indicaram que as estratégias vitais para lidar com a principal barreira são a formação dos profissionais de saúde e a educação continuada^(8,20,21,22,24,25)^. Estima-se que, em média, cinco horas dos cursos de formação de médicos são dedicados à saúde LGBTQIA+, sem um contexto específico à transexualidade, demandando busca autônoma dos profissionais para o aperfeiçoamento das suas habilidades. Essa estimativa é ainda menor em cursos de enfermagem, com duração entre uma e duas horas^(30)^.

Na educação continuada, recomenda-se o enfoque em ensinar relações básicas de gênero, diversidade, experiências e estratégias para um cuidado digno^(8)^. Vale ressaltar que esses treinamentos não devem ser tidos de forma pontual, sem uma atualização posterior; para que a estratégia seja de fato efetiva, recomenda-se uma constância. Outro aspecto é que a educação não deve ser privativa apenas aos profissionais de saúde, pois a RAS é composta por diversas profissões que devem ser também aperfeiçoadas^(20)^. Nesse contexto, a formulação de protocolos institucionais^(22)^ pode ser uma estratégia promotora de uma assistência humanizada e sensível às crianças e aos adolescentes transexuais.

Nos estudos internacionais, observou-se a indicação da cobertura paga e limitada dos seguros de saúde aos cuidados relacionados à transexualidade como uma barreira para o acesso^(22,23)^. No Brasil, as políticas públicas garantem um acesso universal e integral à RAS pelas crianças e adolescentes LGBTQIA+ no SUS de forma gratuita^(17,18,19,22)^, aspecto positivo que deve ter visibilidade, demonstrando a potencialidade de uma rede de saúde pública. Aqui, outros problemas podem ser mais frequentes, como a interligação da RAS e a distância dos serviços de saúde qualificados.

A ausência da interligação entre a RAS, especialmente entre a atenção primária e a atenção especializada, é um desafio da rede de saúde brasileira que vai além da saúde da criança e do adolescente transexual. Esse contexto leva à peregrinação entre serviços, com ausência de um cuidado longitudinal^(21,22)^. Esforços coletivos precisam ser conduzidos para modificar esse cenário, e as estratégias listadas no Quadro 1 podem ser integradas em estudos de implementação para a garantia dessa articulação.

Há também a dificuldade de acesso, seja pelo local de moradia ou pela distância. Uma estratégia indicada nesse contexto é o teleatendimento que, após a pandemia de COVID-19, ganhou maior visibilidade^(23)^. É digno de nota que, mesmo em atendimentos remotos, todas as estratégias citadas no Quadro 1 devem ser integradas para um cuidado acolhedor.

Ao retratarmos esse acesso, é preciso refletir que, para além da transexualidade, com o crescimento das crianças e vivência da adolescência, os serviços de saúde deixam de ser acessados com frequência, com a redução do número de consultas de puericultura na atenção primária, porta de entrada da rede^(19)^. O olhar do profissional se volta à primeira infância, em detrimento das etapas subsequentes, e ao processo de adoecimento, de forma que o adolescente não é visto pelas políticas públicas. No sistema de saúde, as ações se voltam à redução de danos, educação sexual com enfoque na prevenção de infecções sexualmente transmissíveis e gestação, com redução da assistência a funções reprodutivas, deixando de lado toda a singularidade da fase^(3)^. Assim, de forma intrínseca, a RAS deixa de ser um potencial na vida da criança e do adolescente, repercutindo ao longo da fase adulta^(3,9)^. Isso pode ser mais frequente nos casos de transexualidade.

A Carta de Direito dos Usuários de Saúde^(17)^ indica que todo cidadão tem o direito a tratamento adequado e efetivo para sua demanda de cuidado. Todavia, neste estudo, foi observado que a queixa das crianças e adolescentes transexuais tende a ser focalizada no gênero, independentemente da demanda^(22,26)^. Uma ilustração disso são as crianças e adolescentes transexuais que buscam o serviço por agravos respiratórios, mas a equipe de saúde, apesar de tratar dessa queixa, focaliza todo o cuidado no fato de serem transexuais, o que pode potencializar o seu afastamento dos serviços de saúde, especialmente se esse olhar à transexualidade seja marcado de estigma e patologização da identidade de gênero.

A título de ilustração, em investigação inclusa, um adolescente de 16 anos relatou que: “*Estou muito doente, mas não quero ter que lidar com todo o ódio no consultório médico ou toda a discriminação contra mim*”. O mesmo ressalta que não vai à RAS pela ansiedade de que não será aceito no espaço: “*É difícil ir ao médico se você é odiado lá*”^(8)^, reiterando a necessidade do acolhimento. É digno de nota que a transexualidade deve ser a protagonista do cuidado quando a busca pelo serviço for devido a essa e, em outros casos, o atendimento deve ocorrer de forma a respeitar a identidade de gênero, mas atuar na queixa base que levou a criança ou adolescente ao serviço de saúde^(23)^.

Ao fim, a ambiência dos serviços e a homofobia estrutural também foram barreiras identificadas^(20,26)^. O Quadro 1 traz estratégias para a modificação da ambiência, porém cabe ressaltar que essa não é válida, de forma isolada, se o cuidado prestado pelos profissionais de saúde não for acolhedor. Ao promover o ambiente físico preparado para receber crianças e adolescentes transexuais, faz-se necessário que os profissionais estejam capacitados para o atendimento da população.

As estratégias de acesso e acolhimento apresentadas neste estudo demonstram que crianças e adolescentes transexuais não querem ser tratados de maneira diferente ou especial, mas querem que seu atendimento seja como de qualquer outro paciente, sem a necessidade de esconder quem são. Todas as medidas de acolhimento aqui apresentadas podem ser colocadas em prática, desde que o serviço de saúde esteja disposto. Treinamento de equipes, elaboração de protocolos e auditoria frequente deveriam ser implementados em todos os serviços de saúde, desde a atenção primária até os serviços terciários, públicos ou privados. Essas medidas demonstrariam que os funcionários desses serviços de saúde são respeitosos, acolhedores e provedores de um ambiente seguro para qualquer tipo de tratamento^(12)^. Contudo, sabe-se que essas estratégias possuem limitações intrínsecas, como o caso de treinamentos isolados sem uma constância. Assim, indica-se a necessidade de refletir sobre uma intersecção entre as inúmeras estratégias indicadas neste artigo e o contexto de atuação, de forma a aumentar a chance de sucesso na prática clínica.

### Limitações do estudo

Este estudo possui como limitação a centralização de estudos qualitativos, o que não permite demonstrar a magnitude dos impactos das barreiras e estratégias indicadas. Contudo, esse aspecto não reduz a potencialidade dos dados. Na política LGBTQIA+, indica-se como diretriz a necessidade da produção de conhecimentos científicos^(12)^, com este estudo sendo incluso nesse aspecto.

### Contribuições para a área da saúde

Espera-se que esta revisão contribua para a reorientação e/ou construção de diretrizes ou políticas públicas que visem garantir o acesso e acolhimento às crianças e aos adolescentes transexuais em uma RAS interligada e otimizada, para reconhecer, validar e acolher sua identidade de gênero, com cuidados apropriados, seguros e inclusivos.

## CONCLUSÕES

Este estudo demonstrou as barreiras vivenciadas por crianças e adolescentes transexuais na RAS e as estratégias para superá-las. Em oito estudos, observou-se que as principais barreiras foram baixo conhecimento profissional, interações profissionais hostis e presunção sexual. As estratégias foram o questionamento da identidade de gênero, desde a admissão na RAS, e a educação continuada de profissionais de saúde. Espera-se que, com a demonstração deste contexto, os profissionais de saúde possam refletir suas práticas, de forma a minimizar os impasses e potencializar um acesso universal e acolhedor para as crianças e adolescentes transexuais, com a interligação das estratégias supracitadas na sua assistência, além de caminhar em estudos de implementação que possibilitem a tradução das políticas públicas existentes à prática clínica, de forma a respeitar os direitos das crianças e dos adolescentes transexuais.
